# The genetic architecture of leaf number and its genetic relationship to flowering time in maize

**DOI:** 10.1111/nph.13765

**Published:** 2015-11-23

**Authors:** Dan Li, Xufeng Wang, Xiangbo Zhang, Qiuyue Chen, Guanghui Xu, Dingyi Xu, Chenglong Wang, Yameng Liang, Lishuan Wu, Cheng Huang, Jinge Tian, Yaoyao Wu, Feng Tian

**Affiliations:** ^1^ National Maize Improvement Center of ChinaChina Agricultural UniversityBeijing100193China

**Keywords:** flowering time, genetic overlap, leaf number, maize (*Zea mays*), quantitative trait locus (QTL)

## Abstract

- The number of leaves and their distributions on plants are critical factors determining plant architecture in maize (*Zea mays*), and leaf number is frequently used as a measure of flowering time, a trait that is key to local environmental adaptation.
- Here, using a large set of 866 maize‐teosinte BC_2_S_3_ recombinant inbred lines genotyped by using 19 838 single nucleotide polymorphism markers, we conducted a comprehensive genetic dissection to assess the genetic architecture of leaf number and its genetic relationship to flowering time.
- We demonstrated that the two components of total leaf number, the number of leaves above (LA) and below (LB) the primary ear, were under relatively independent genetic control and might be subject to differential directional selection during maize domestication and improvement. Furthermore, we revealed that flowering time and leaf number are commonly regulated at a moderate level. The pleiotropy of the genes *ZCN8*,* dlf1* and *ZmCCT* on leaf number and flowering time were validated by near‐isogenic line analysis. Through fine mapping, *qLA1‐1*, a major‐effect locus that specifically affects LA, was delimited to a region with severe recombination suppression derived from teosinte.
- This study provides important insights into the genetic basis of traits affecting plant architecture and adaptation. The genetic independence of LA from LB enables the optimization of leaf number for ideal plant architecture breeding in maize.

## Introduction

The number of leaves and the proportion of leaves above and below the primary ear are critical determinants of plant architecture in maize. The total leaf number (TLN) of a maize plant consists of two components, the number of leaves above the primary (uppermost) ear (LA) and the number of leaves below the primary ear (LB). An optimal proportion of LA and LB is critical for the development of ideal plant architecture to improve plant population structure and enhance photosynthetic efficiency. Despite its importance, the way in which maize leaf number is genetically controlled remains largely unknown. Our current knowledge of leaf number in maize is based mainly on genetic studies of the highly correlated trait, flowering time, that is key to local environmental adaptation. A comprehensive genetic study is thus needed to dissect the genetic architecture of maize leaf number and to identify its genetic relationship to flowering time.

Leaves are sequentially initiated from the shoot apical meristem (SAM) in repeating modules known as phytomers before plants enter reproductive growth (Langdale, 2005; Colasanti & Muszynski, 2009). Each phytomer consists of a leaf, axillary meristem (AM) and internode (Langdale, 2005). The number of phytomers or leaves required before the reproductive phase change is determined largely by the rate and pattern of leaf initiation during vegetative development and the duration of the vegetative phase controlled by the timing of floral transition (Langdale, 2005; Colasanti & Muszynski, 2009). Several genes that affect the rate and pattern of leaf initiation have been cloned, and mutations in these genes usually alter the spatial (phyllotaxy) and temporal (plastochron) patterns of leaf initiation, frequently in association with changes in leaf number (Veit *et al*., 1998; Jackson & Hake, 1999; Giulini *et al*., 2004; Chuck *et al*., 2010, 2014). For example, the *SBP‐box* transcription factors *unbranched2* (*ub2*), *unbranched3* (*ub3*) and *tasselsheath4* (*tsh4*) function as redundant factors that limit the rate of cell differentiation to the lateral domains of meristems. In addition to affecting inflorescence structures, their double or triple mutants have an increased number of leaves because of the shortened plastochron (Chuck *et al*., 2014). When plants switch from vegetative to reproductive development, the SAM ceases leaf initiation and becomes committed to the production of reproductive structures (McSteen *et al*., 2000). Therefore, the total number of leaves produced by a plant is fixed by the floral transition and has been frequently used as a measure of flowering time in maize.

Significant advances in understanding the molecular mechanisms underlying flowering time have been made, primarily from studies in *Arabidopsis* and rice (Shrestha *et al*., 2014; Blümel *et al*., 2015). By contrast, the genetic control of flowering time in maize has been less extensively studied. Several late‐flowering mutants of maize with delayed floral transition have been characterized and all produce more leaves than wild‐type plants (Colasanti & Muszynski, 2009). *Leafy* (unrelated to the *Arabidopsis LEAFY* gene) is a dominant, late‐flowering mutation that specifically increases the number of leaves above the uppermost ear (Neuffer *et al*., 1997) but has not been molecularly isolated. *Indeterminate1* (*id1*) encodes a zinc finger transcription factor and is expressed mainly in the immature leaf before and after the floral transition. *id1* is specific to monocots and plays a central role in the endogenous pathway of maize flowering time regulation (Colasanti *et al*., 2006). *Delayed flowering1* (*dlf1*), a basic Leu zipper (bZIP) transcription factor, is another gene that is required in the shoot apex for the proper timing of the floral transition in maize and functions downstream of *id1* (Muszynski *et al*., 2006). Functional analyses of a set of *FLOWERING LOCUS T* (*FT*)‐like genes in maize identified *ZEA CENTRORADIALIS 8* (*ZCN8*) as a florigen. *ZCN8* is transcribed and translated in leaves and then moves via the phloem to the SAM where it interacts with the DLF1 protein to promote the transition from vegetative to reproductive development (Meng *et al*., 2011). Upregulating and downregulating *ZCN8* mRNA also causes changes in leaf number. Comparative genomics and mutant analyses have identified several other flowering time genes in maize (Sheehan *et al*., 2007; Danilevskaya *et al*., 2008; Miller *et al*., 2008; Dong *et al*., 2012).

Maize exhibits substantial natural variation in leaf number, with a total number of leaves ranging from seven to 19 leaves in a panel with diverse maize inbred lines (Flint‐Garcia *et al*., 2005). However, few studies have dissected the genetic basis of natural variation in maize leaf number (Vlăduţu *et al*., 1999; Wang *et al*., 2008). By contrast, genetic studies of maize flowering time have been conducted intensively in biparental populations (e.g. Koester *et al*., 1993; Beavis *et al*., 1994; Austin & Lee, 1996; Ribaut *et al*., 1996; Veldboom & Lee, 1996; Vlăduţu *et al*., 1999; Moutiq *et al*., 2002; Salvi *et al*., 2002; Balint‐Kurti *et al*., 2007; Briggs *et al*., 2007; Szalma *et al*., 2007) and recently have been extended to multiple‐line crosses and diverse association panels, enabling higher resolution identification of quantitative trait loci (QTL) and a better understanding of the genetic architecture of flowering time (Chardon *et al*., 2004; Wang *et al*., 2008; Buckler *et al*., 2009; Coles *et al*., 2010; Huang *et al*., 2012, 2015; Steinhoff *et al*., 2012). Although a large number of flowering time QTLs have been mapped in maize, only *Vegetative to generative transition 1* (*Vgt1*) and *ZmCCT* have been successfully cloned. *Vgt1*, a QTL for leaf number and flowering time on chromosome 8 (Phillips *et al*., 1992; Vlăduţu *et al*., 1999; Salvi *et al*., 2002), was narrowed down to a *c*. 2‐kb noncoding region that regulates an APETALA2‐like gene (*ZmRap2.7*) located 70 kb downstream (Salvi *et al*., 2007). *ZmCCT*, a homologue of the rice photoperiod response regulator *Ghd7* (Xue *et al*., 2008), is another recently cloned gene that affects photoperiod response in maize (Ducrocq *et al*., 2009; Hung *et al*., 2012). The insertion of a CACTA‐like transposable element in the promoter of *ZmCCT* results in early flowering and a decrease in leaf number, and was selected during the postdomestication adaptation of maize to longer days (Yang *et al*., 2013).

The association of these key flowering time genes with changes in leaf number suggests that flowering time and leaf number are commonly regulated in maize. However, population‐level phenotypic analysis has indicated that the phenotypic correlation between flowering time and leaf number is far from complete, suggesting genetic heterogeneity between leaf number and flowering time in maize. A comprehensive genetic study is required to determine the extent of genetic sharing between leaf number and flowering time in maize. In this study, using a large set of 866 maize‐teosinte BC_2_S_3_ recombinant inbred lines (RILs) previously genotyped by using 19 838 single nucleotide polymorphism (SNP) markers (Shannon, 2012), we conducted high‐resolution QTL mapping for leaf number and flowering time and investigated their genetic overlap. Our results showed the following: the two components of total leaf number (LA and LB) are controlled by contrasting genetic architectures and tend to be under relatively independent genetic control; LA and LB may be under differential directional selection during maize domestication and improvement; flowering time and leaf number exhibit a moderate level of genetic sharing, primarily as a result of shared loci between flowering time and LB rather than LA; *qLA1‐1* is a major‐effect locus that specifically affects the number of leaves above the primary ear and is located in a region with significant recombination suppression derived from teosinte.

## Materials and Methods

### Materials

A large population including 866 BC_2_S_3_ RILs was obtained from the Maize Coop Stock Center. This population was derived from a cross between W22 (a typical temperate maize inbred line) and CIMMYT accession 8759 (a typical accession of *Zea mays* ssp. *parviglumis*). Detailed information about the BC_2_S_3_ RIL population is provided in Shannon (2012) and Huang *et al*. (2015). The 866 maize‐teosinte BC_2_S_3_ RILs were previously genotyped by using 19 838 SNPs (Shannon, 2012). Owing to the large sample size and high‐density markers, this population has been used as a powerful tool for trait dissection and gene cloning (Hung *et al*., 2012; Lin *et al*., 2012; Wills *et al*., 2013; Lang *et al*., 2014; Huang *et al*., 2015).

### Field trials and phenotyping

The maize‐teosinte BC_2_S_3_ RILs were planted at Shangzhuang Experimental Station of China Agricultural University, Beijing (39.9°N, 116.4°E), China, in the summers of 2012 and 2013. In each trial, the 866 BC_2_S_3_ RILs were grown in a single replicate employing an augmented incomplete randomized blocks design widely used to phenotype large populations (Buckler *et al*., 2009; Kump *et al*., 2011; Tian *et al*., 2011; Huang *et al*., 2015). Two maize inbred lines W22 and Mo17 were randomly inserted in each incomplete block as checks. Each line was grown in a three‐row plot in 2012 and a single‐row plot in 2013, with 15 plants per row, 25 cm between plants within each row and 50 cm between rows.

In order to measure the number of leaves of each RIL, the 5^th^ and 10^th^ leaf of five consecutive plants from the middle of each plot were marked. The LA and LB were counted for each individual plant. The TLN was scored as the sum of LA and LB. For each line, the days to anthesis (DTA) for the same set of individual plants scored for leaf number were recorded. The average of the phenotypic values of the five sampled plants was used as the phenotype of each RIL in each trial.

### Phenotypic data analysis

Following the previously described procedure to minimize the effects of environment variation (Buckler *et al*., 2009; Kump *et al*., 2011; Tian *et al*., 2011; Huang *et al*., 2015), the 2 yr of phenotypic data were fitted with a linear mixed model that included the effects of years, genotypes, incomplete blocks, field columns and field rows. In the mixed model, spatial correlations among residuals were modelled with first‐order autoregressive (AR1 × AR1) residual structures to further account for potential field spatial variation (Gilmour *et al*., 1997). Likelihood ratio tests were used to determine which effects to retain in the final model. The best linear unbiased predictor (BLUP) for each line was predicted with ASREML (Gilmour *et al*., 2009) and used as the phenotypic input in subsequent QTL mapping.

### QTL mapping

The QTL mapping method was the same as that described in Shannon (2012) and Huang *et al*. (2015). Briefly, a modified version of R/qtl (Broman *et al*., 2003) that takes into account the BC_2_S_3_ pedigree of the RILs was used for QTL mapping (Shannon, 2012). A multiple QTL mapping procedure was used to identify QTLs. The *scanone* command in R/qtl that employs simple interval mapping using Haley–Knott regression was first conducted (Broman *et al*., 2003). A total of 10 000 permutations of the phenotypic data were performed for each trait to determine a *P *<* *0.05 logarithm of odds (LOD) significance threshold level. The QTL list from *scanone* scanning was then used as a starting point for subsequent multiple QTL fitting. Each model was confirmed using a drop‐one ANOVA, such that only the QTLs with a LOD score greater than the threshold and an ANOVA *P*‐value < 0.05 were retained. To further refine the position of each QTL, the *refineqtl* command was then used by fitting all QTLs in the model. The likelihood ratio test was used to measure the improvement of the model. Finally, the *addqtl* tool was used to search additional QTLs to improve the model. If a new QTL was added, the ANOVA and *refineqtl* procedure were repeated to evaluate the fit of the new model. The entire process was repeated until no more significant QTLs could be added. The total phenotypic variation explained by all QTLs was calculated from a full model that fitted all QTL terms in the model using a *fitqtl* function. The percentage of phenotypic variation explained by each QTL was estimated using a drop‐one‐ANOVA analysis implemented in a *fitqtl* function. The confidence interval for each QTL was defined using a 2‐LOD support interval. To investigate the genetic overlap among TLN, LA, LB and DTA, the 2‐LOD support intervals of identified QTLs were compared, and QTLs with overlapping support intervals were considered common QTLs for the compared traits.

### QTL correspondence between different traits

In order to evaluate the significance of correspondence of QTLs for leaf number and flowering time traits, a previously described statistical test based on the hypergeometric probability distribution (Lin *et al*., 1995; Feltus *et al*., 2006) was used to calculate the probability of obtaining the observed number of matching QTL by chance alone. The equation is as follows:

$$P=\frac{\left(\begin{array}{c} l\\ m \end{array}\right)\;\left(\begin{array}{c} n-l\\ s-m \end{array}\right)}{\left(\begin{array}{c} n\\ s \end{array}\right)}$$

(*n*, total number of comparison intervals). The total map length of BC_2_S_3_ population is 1488 cM and the average interval size of mapped QTLs is *c*. 8 cM. This yields *n *=* *186 intervals over which correspondence of QTLs can be compared in this study. *m* is the number of matches declared between two compared traits. *l* and *s* are the larger and smaller number of QTLs detected for the two compared traits, respectively.

### QTL effect validation and fine mapping

In order to validate the phenotypic effects of several selected target QTLs, following a strategy described previously (Huang *et al*., 2015), near‐isogenic lines (NILs) were developed from a heterogeneous inbred family (HIF) that is only heterozygous at the target QTL (Tuinstra *et al*., 1997). For each target QTL, a small HIF‐derived F_2_ population with > 150 plants was planted in Sanya (18°N, 109°E), Hainan province, China, in the winter of 2013. Within each segregating family of target QTLs, individual plants that were homozygous for W22 across the target region, designated as NIL^maize^, and plants that were homozygous for teosinte, designated as NIL^teosinte^, were identified by markers and selfed for progeny phenotype testing. For each target QTL, the paired NIL^maize^ and NIL^teosinte^ were planted in neighbouring rows and scored for LA, LB, TLN and DTA in the same field in the summer of 2014 in Beijing. A *t*‐test was used to compare the phenotypic difference between NIL^maize^ and NIL^teosinte^ at *P *<* *0.01.

In order to fine map *qLA1‐1*, a large F_2_ population derived from a HIF (Supporting Information Fig. S1) was planted in the winter nursery in Hainan province in 2013. Recombinants were identified using markers across the target region. Along with the QTL NIL trial described earlier, the recombinant derived F_3_ families were planted in the same field in Beijing in the summer of 2014. A similar within‐family comparison strategy (Hung *et al*., 2012; Huang *et al*., 2015) was used to delimit the region of *qLA1‐1*. Briefly, in the recombinant‐derived F_3_ family, homozygous recombinant (HR) and homozygous nonrecombinant (HNR) plants were identified using appropriate markers. Phenotypic differences were tested between HR and HNR pairs within each family. If there was a significant phenotypic difference between the HR and HNR pairs, the parental F_2_ recombinant was heterozygous for the target QTL; otherwise, it was homozygous for either parent. *T*‐tests with Bonferroni corrections for multiple testing were used to test the phenotypic differences between HR and HNR pairs (*P *<* *0.01). A substitution mapping procedure (Paterson *et al*., 1990) was used to delimit the causal QTL region.

### Molecular analyses

DNA was extracted from fresh leaves using the CTAB method (Murray & Thompson, 1980) with minor modifications. To fine map *qLA1‐1*, InDel markers were developed based on the B73 reference genome (B73 RefGen_v2) (http://www.maizegdb.org/). Primers were designed using software Primer 3.0 (http://bioinfo.ut.ee/primer3-0.4.0/). The PCR system was the same as that previously described (Huang *et al*., 2015). Sequence alignments were performed with BioEdit (v.7.0.9.0, North Carolina State University, Raleigh, NC, USA) and manually edited if necessary. To facilitate population genotyping, only sequences that showed large insertion or deletion polymorphisms between NILs and that could be well determined electrophoretically on 3% agarose gel were further developed into InDel markers.

## Results

### Phenotypic analysis

**Figure 1:**
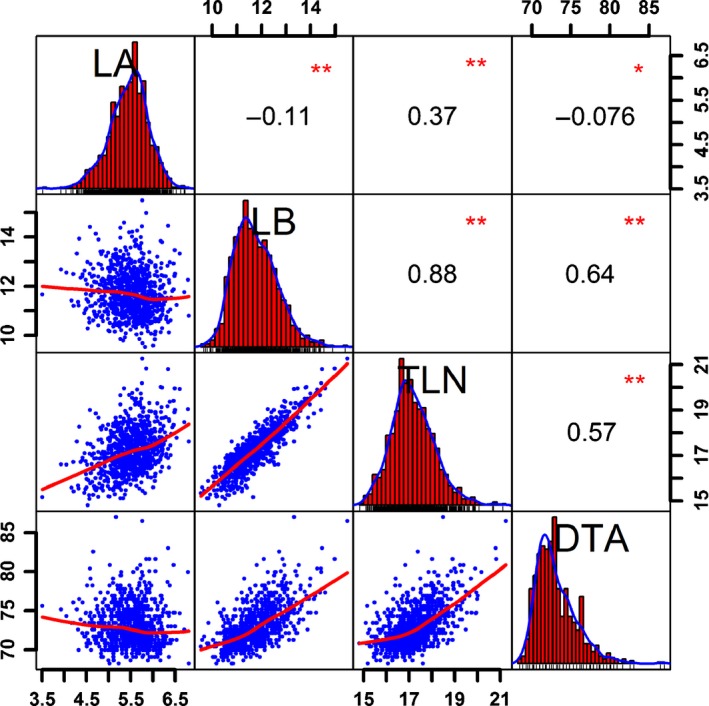
Phenotypic analysis of leaf number and flowering time in the maize‐teosinte BC_2_S_3_ recombinant inbred line population. The plots on the diagonal show the phenotypic distribution of each trait. The values above the diagonal are pairwise correlation coefficients between traits*,* and the plots below the diagonal are scatter plots of compared traits. *, *P *<* *0.05; **, *P *<* *0.01. LA, the number of leaves above the primary ear; LB, the number of leaves below the primary ear; TLN, total leaf number; DTA, days to anthesis.

As shown in Fig. 1, the maize‐teosinte BC_2_S_3_ RIL population exhibited wide phenotypic variations in the four traits of leaf number and flowering time, and all phenotypic correlations among the four traits reached significance at *P *<* *0.01, but the degree of correlation varied substantially among the traits. The two leaf number components, LA and LB, exhibited a relatively low negative correlation (*r *=* *−0.11), whereas the phenotypic correlation between DTA and TLN was relatively high (*r *=* *0.57), mostly owing to the high phenotypic correlation between DTA and LB (*r *=* *0.64), the major component of TLN. The phenotypic correlation between DTA and LA was low (*r *=* *−0.076).

### QTLs for leaf number and flowering time

Table 1 summarizes the QTL mapping results for LA, LB, TLN and DTA and the underlying genetic architecture features. The full QTL list for the four traits is presented in Table S1.

**Table 1:** Summary of quantitative trait loci (QTLs) identified for four leaf number and flowering time traits in the maize‐teosinte BC_2_S_3_ population

Traits	Number of QTLs	% Variation explained by all QTLs	% Variation explained by each QTL	Genetic architecture feature
LA	15	65.4	1.0–21.5	A major‐effect QTL plus many small‐effect QTLs
LB	19	70.0	1.0–6.0	Numerous small‐effect QTLs
TLN	20	69.3	0.9–9.8	Numerous small‐effect QTLs
DTA	19	66.3	1.1–16.6	A major‐effect QTL plus many small‐effect QTLs

LA, the number of leaves above the primary ear; LB, the number of leaves below the primary ear; TLN, total leaf number; DTA, days to anthesis.

**Figure 2:**
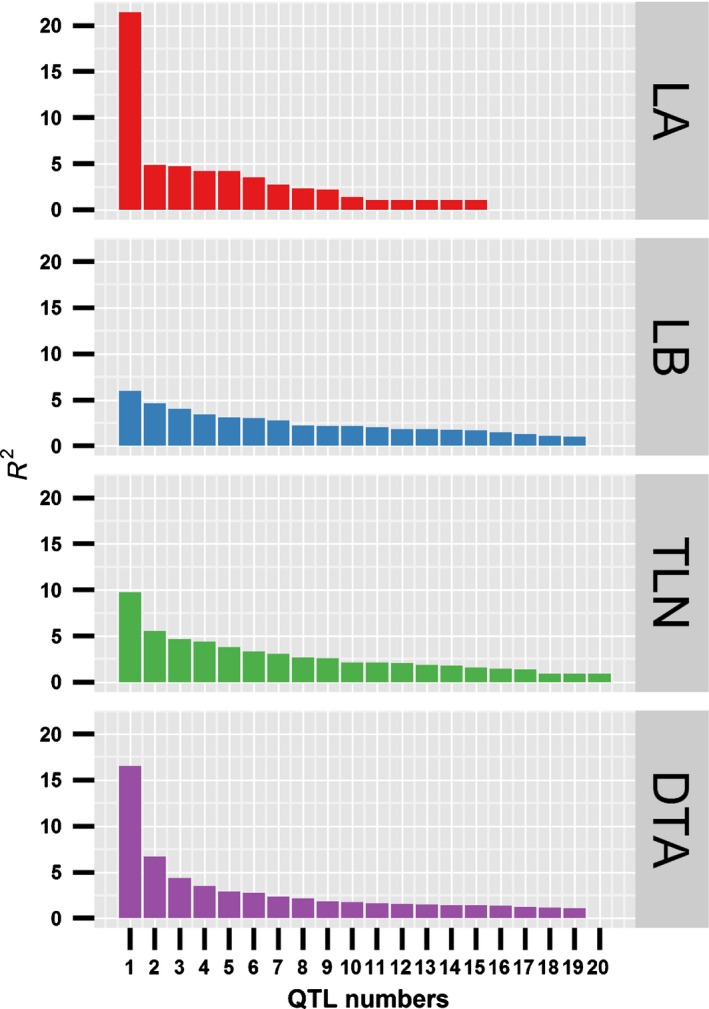
The effect size of quantitative trait loci (QTLs) for leaf number and flowering time traits in the maize‐teosinte BC_2_S_3_ population. The *x*‐axis indicates the number of QTLs and the *y*‐axis indicates the phenotypic variation explained by each QTL (*R* ^2^). LA, the number of leaves above the primary ear; LB, the number of leaves below the primary ear; TLN, total leaf number; DTA, days to anthesis.

For LA, a total of 15 QTLs were identified (Tables 1, S1; Fig. 2). These QTLs jointly explained 65.4% of the total phenotypic variation of LA (Table 1). The phenotypic variation explained by each QTL ranged from 1% (*qLA1‐3*,* qLA8‐2*,* qLA9‐1* and *qLA10‐1*) to 21.5% (*qLA1‐1*) (Table 1). In particular, *qLA1‐1* on chromosome 1 is a major‐effect locus for LA, with an LOD value of 89.8. The teosinte allele at *qLA1‐1* resulted in 0.42 fewer leaves above the primary ear. Except *qLA1‐1*, no QTL explained > 5% of the phenotypic variation (Fig. 2). This result suggests that LA is controlled by a large‐effect QTL plus many small‐effect QTLs.

For LB, a total of 19 QTLs were detected that jointly explained 70% of the total phenotypic variation (Table 1; Fig. 2). Each QTL for LB explained 1.0% (*qLb2‐3*) to 6.0% (*qLb7‐1*) of the total phenotypic variation. In contrast to LA, no QTL for LB explained > 10% of the phenotypic variation (Fig. 2), suggesting that the genetic architecture of LB is relatively more complex and is controlled by a large number of small‐effect QTLs.

For TLN which was scored as the sum of LA and LB, a total of 20 QTLs were identified that jointly explained 69.3% of the phenotypic variation (Table 1; Fig. 2). The phenotypic variation explained by each TLN QTL ranged from 0.9% (*qTLN3‐2*) to 9.8% (*qTLN8‐1*). Although a large‐effect QTL (*qLA1‐1*) was detected for LA, it explained only 5.5% of the phenotypic variation of TLN. None of the TLN QTLs explained > 10% of the phenotypic variation (Fig. 2), suggesting the complexity of the genetic architecture of TLN as a joint trait of LA and LB.

For DTA, a total of 19 QTLs were mapped that jointly explained 66.3% of the phenotypic variation of DTA. The phenotypic variation explained by each QTL ranged from 1.1% (*qDTA6‐3*) to 16.6% (*qDTA10‐1*). Except *qDTA10‐1* on chromosome 10, no QTL could explain > 10% of the phenotypic variation (Fig. 2), suggesting that DTA is controlled mainly by a relatively large‐effect QTL plus many small‐effect QTLs.

### Independent genetic control and differential directional selection for LA and LB

**Figure 3:**
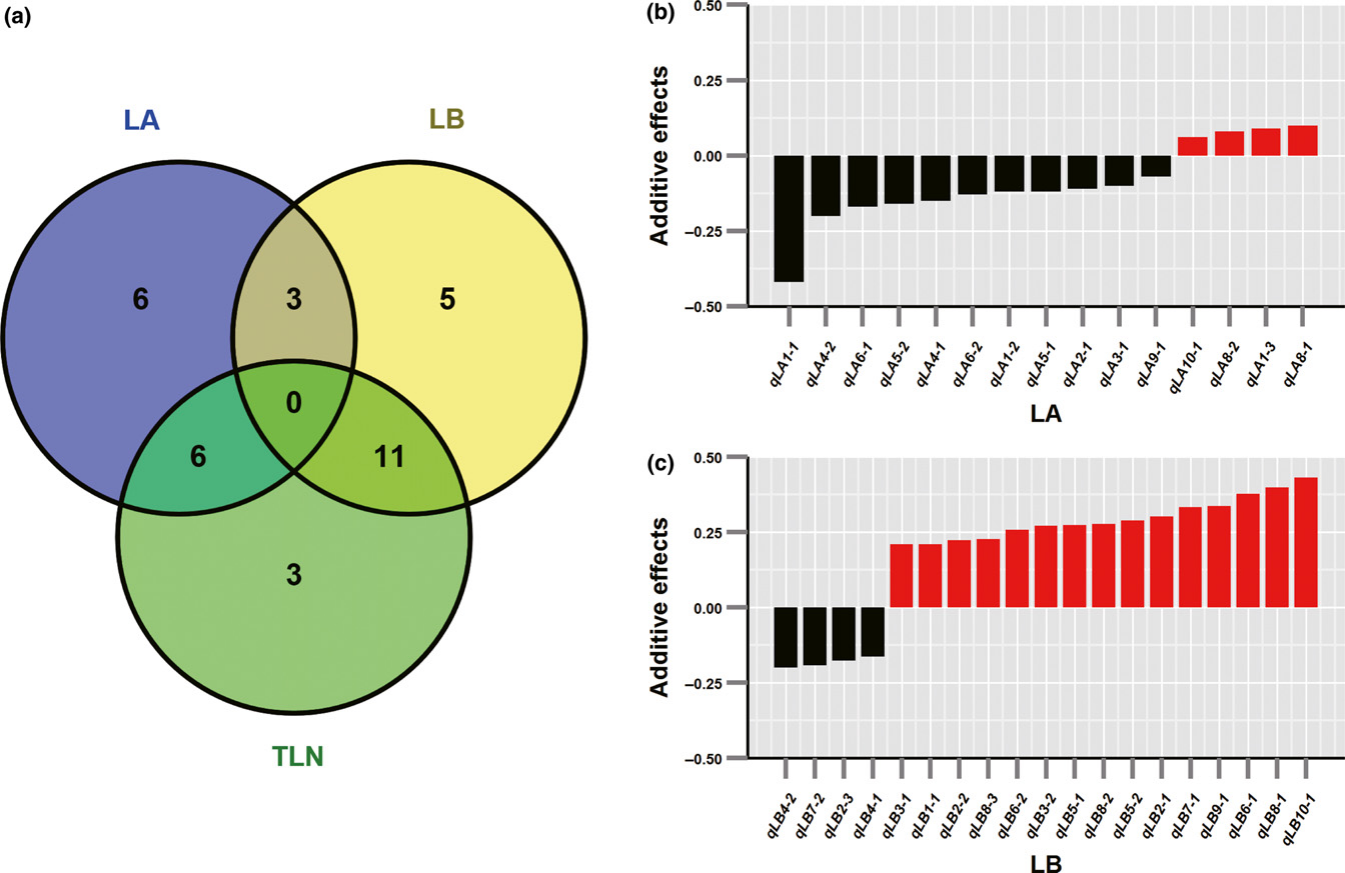
The genetic relationships among leaf number traits in the maize‐teosinte BC2S3 population. (a) The quantitative trait locus (QTL) overlap among the number of leaves above the primary ear (LA), the number of leaves below the primary ear (LB) and total leaf number (TLN). The additive effect distribution of QTLs for (b) LA and (c) LB. The *x*‐axis indicates the name of each QTL, and the *y*‐axis indicates the additive effect of the teosinte allele relative to the maize allele at each QTL.

Figure 3(a) shows the overlap among QTLs for the three leaf number traits (LA, LB and TLN). The associated significances of QTL correspondence between compared traits are included in Table S2. Of 15 LA QTLs, six (40%) also showed effects on TLN (*P *=* *0.0017, Table S2). Of 19 LB QTLs, 11 (58%) also showed significant effects on TLN (*P *=* *4.83E‐08, Table S2). However, only three QTLs (9.7% of all LA and LB QTLs) were simultaneously detected for LA and LB. This overlap is not significantly different from expected by chance alone (*P *=* *0.1344, Table S2), suggesting that LA and LB might be under relatively independent genetic control. This result could explain the relatively low phenotypic correlation between LA and LB observed in the previous phenotypic analysis. Furthermore, at the three common QTLs for LA and LB (*qLA5‐1* and *qLB5‐1*;* qLA2‐1* and *qLB2‐2*; and *qLA6‐1* and *qLB6‐2*), the parental alleles exhibited opposite effect directions: teosinte alleles decreased LA but increased LB. This opposite parental contribution could explain why no significant TLN effects were detected at these three common loci for LA and LB. These results suggest the need to further partition the TLN trait to avoid missing QTLs that are specific to the LA or LB component trait.

The analysis of the positive or negative effect of QTL revealed that the teosinte alleles at 11 (73.3%) of 15 QTLs for LA were associated with the effect of decreasing the number of leaves above the primary ear (Fig. 3b). However, in contrast to the allelic effect distribution of LA QTLs, the teosinte alleles at 15 (78.9%) of 19 QTLs for LB tended to increase the number of leaves below the primary ear (Fig. 3c). This contrasting QTL allele effect direction for LA and LB suggests that the two components of leaf number were under differentially directional selection during maize domestication and improvement.

### Genetic overlap between leaf number and flowering time

**Figure 4:**
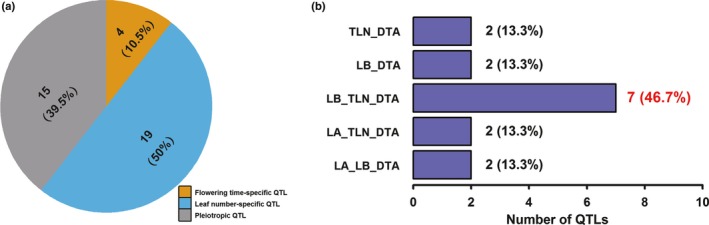
The genetic overlap between leaf number and flowering time in the maize‐teosinte BC_2_S_3_ population. (a) Quantitative trait locus (QTL) categories and their proportions. (b) The pleiotropic effect patterns across the four leaf number and flowering time traits for the 15 shared loci. LA, the number of leaves above the primary ear; LB, the number of leaves below the primary ear; TLN, total leaf number; DTA, days to anthesis.

In order to evaluate the genetic overlap between leaf number and flowering time, the 2‐LOD support intervals of QTLs for LA, LB, TLN and DTA were compared. Based on their overlaps, all QTLs were classified into three categories corresponding to 38 nonoverlapping regions in the maize genome (Table 2). Of these 38 regions, four (10.5%) loci affected only DTA, 19 (50%) loci were specific to leaf number, and 15 (39.4%) regions exhibited pleiotropic effects for leaf number and DTA (Fig. 4a), suggesting that leaf number and flowering time are commonly regulated at a modest level. If considering DTA individually, of 19 DTA QTLs, 15 (79%) simultaneously showed effects on leaf number. However, if considering leaf number individually, of 34 nonoverlapping leaf number QTLs, only 15 (44%) were also associated with effects on DTA. This contrasting result suggests that QTLs for DTA are more likely simultaneously accompanied by changes in leaf number, as frequently observed in previous mutant analyses of several flowering time genes (Colasanti *et al*., 2006; Muszynski *et al*., 2006). By contrast, QTLs for leaf number are not always associated with changes in flowering time.

**Table 2:**
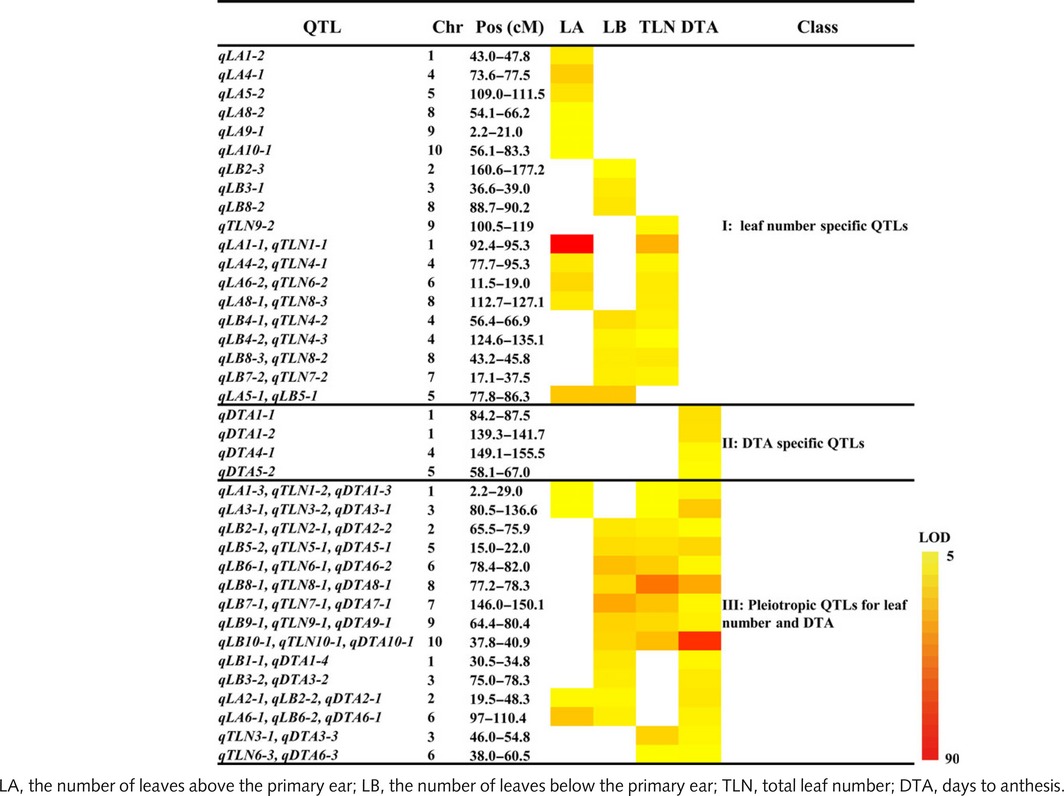
Genetic overlap between leaf number and flowering time traits in the maize‐teosinte BC_2_S_3_ population

For the 15 shared loci for leaf number and flowering time, their pleiotropic effect patterns across the four traits can be classified into five types: LA_LB_DTA; LA_TLN_DTA; LB_TLN_DTA; LB_DTA; and TLN_DTA (Fig. 4b). Of these pleiotropic loci, 11 (73.3%) (7 LB_TLN_DTA, 2 LB_DTA and 2 LA_LB_DTA) involved effects from LB, whereas only four (26.7%) loci involved effects from LA, suggesting that the pleiotropy more likely occurred between DTA and LB than between DTA and LA. This trend is also detected in the individual analyses of QTL correspondence between LA, LB and DTA, in which very significant QTL correspondence was detected between LB and DTA (*P *=* *2.28E‐08; Table S2), whereas the QTLs for LA and DTA were only marginally significantly overlapped (*P *=* *0.0414). These results could explain why LB and DTA exhibited higher phenotypic correlation than LA and DTA.

### Co‐localization with selected regions

Using a population genomics approach, Hufford *et al*. (2012) identified a list of 1179 regions that are likely to be under strong selection during maize domestication and improvement. We determined whether the QTLs for leaf number and flowering time traits overlapped with any previously identified selection features (Table S1). Using a permutation test, we compared the degree of overlap between QTL support intervals and the selection features relative to the amount of overlap expected by chance. The results showed that, for flowering time, the QTL support intervals overlapped with selected regions significantly more than would be expected by chance (*P *<* *0.01), whereas for the three traits of leaf number, the QTL support intervals overlapped with selected regions at a less significant level (*P *<* *0.05). These results suggested that many of the QTLs identified in this study might be important targets of selection during maize domestication and improvement.

### Candidate genes for leaf number and flowering time QTLs

**Figure 5:**
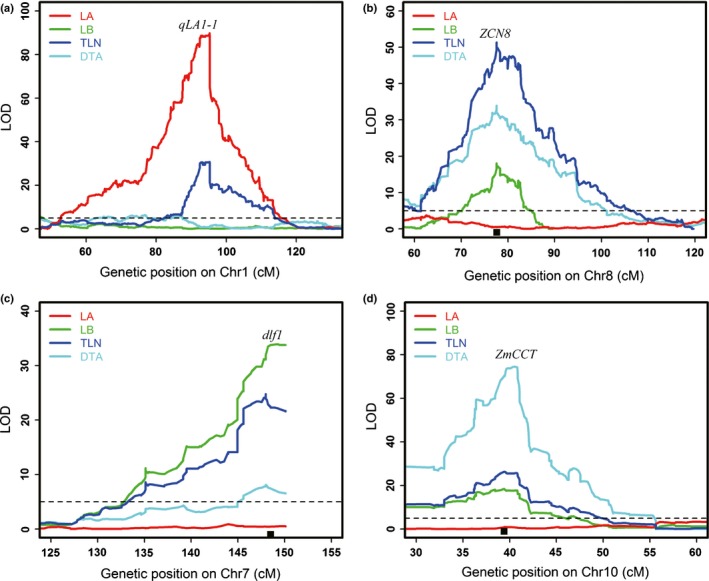
Candidate genes for four leaf number quantitative trait loci (QTLs) mapped in the maize‐teosinte BC_2_S_3_ population. (a) *qLA1‐1*. (b) *qLB7‐1*. (c) *qLB8‐1*. (d) *qLB10‐1*. For each panel, the *x*‐axis shows the genetic position along the chromosomes and the *y*‐axis represents the logarithm of odds (LOD) score of each scanning position. Different coloured lines indicate different traits. The dotted line represents the *P* < 0.05 LOD significance threshold. The black square boxes above the *y*‐axis are the positions of known genes (*dlf1*,*ZCN8* and *ZmCCT*). LA, the number of leaves above the primary ear; LB, the number of leaves below the primary ear; TLN, total leaf number; DTA, days to anthesis.

Through mutant analysis, comparative genomics and QTL cloning, previous studies have identified several important genes that control flowering time in maize (Bomblies *et al*., 2003; Colasanti *et al*., 2006; Muszynski *et al*., 2006; Salvi *et al*., 2007; Meng *et al*., 2011; Dong *et al*., 2012). The mutations at these genes are usually associated with changes in leaf number. We evaluated whether these key flowering time genes underlie the QTLs for DTA in this maize‐teosinte population and also colocalize with QTLs for leaf number traits. As shown in Fig. 5(b–d), we found that the *ZmCCT*,* ZCN8* and *dlf1* genes are all under the peaks of QTLs for DTA (*qDTA10‐1*,* qDTA8‐1* and *qDTA7‐1*), QTLs for LB (*qLB10‐1*,* qLB8‐1* and *qLB7‐1*) and QTLs for TLN (*qTLN10‐1*,* qTLN8‐1* and *qTLN7‐1*), respectively. No significant LA effects were detected at these QTLs. Additionally, *ZAP1*, a MADS box transcription factor that has been previously shown to reduce the number of nodes when ectopically expressed (Heuer *et al*., 2001), is located at the peak of *qLB2‐3* that specifically affects LB (Fig. S2).

### Validation of QTL effects by NILs

**Figure 6:**
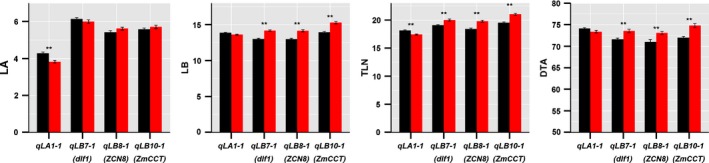
Phenotypic effect analyses of near‐isogenic lines (NILs) for four quantitative trait loci (QTLs) (*qLA1‐1*,*qLB7‐1*,*qLB8‐1* and *qLB10‐1*) mapped in the maize‐teosinte BC_2_S_3_ population. The red and black bars represent NIL ^teosinte^ and NIL ^maize^ at each QTL, respectively. The phenotypic values are shown as the mean ± SE (*n *>* *50) in NIL ^teosinte^ and NIL ^maize^. LA, the number of leaves above the primary ear; LB, the number of leaves below the primary ear; DTA, days to anthesis. **, *P *<* *0.01.

In order to further validate the QTL effects, beginning with the HIF that only segregates for target QTLs, NILs carrying the W22 allele (NIL^maize^) and the teosinte allele (NIL^teosinte^) at the LA QTL (*qLA1‐1*) and the LB QTLs (*qLB10‐1*,* qLB8‐1* and *qLB7‐1*) were developed and scored for flowering time and leaf number traits. At the LB QTLs (*qLB10‐1*,* qLB8‐1* and *qLB7‐1*), *ZmCCT*,* ZCN8* and *dlf1* are the most likely underlying genes (Fig. 5b–d). As shown in Fig. 6, significant phenotypic differences in DTA, LB and TLN were detected between NIL^maize^ and NIL^teosinte^ at *qLB10‐1*,* qLB8‐1* and *qLB7‐1*, whereas no significant phenotypic differences in LA were detected for these three QTLs, consistent with the QTL mapping results. By contrast, at *qLA1‐1*, NIL^maize^ and NIL^teosinte^ only exhibited significant phenotypic difference in LA and TLN, and no significant effects for DTA and LB were detected. This result further suggests that *qLA1‐1* only specifically affects LA without affecting LB and DTA.

### Fine mapping of *qLA1‐1*

**Figure 7:**
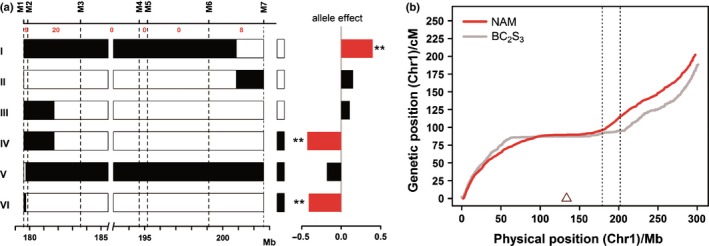
Fine mapping of *qLA1‐1*. (a) Delimiting the position of *qLA1‐1* with recombinants. A total of 37 recombinants were identified using markers from the heterogeneous inbred family derived F_2_ populations. The number of recombinants between markers is shown at the top. The recombinants identified in the F_2_ population were grouped into six groups based on their genotypes. The graphical genotypes of the identified homozygous recombinants (HR) at the target region within each recombinant group are shown on the left. Black or white boxes next to the HR indicate the corresponding homozygous nonrecombinants (HNR) identified within each recombinant family. Black and white boxes indicate homozygous regions for the maize and teosinte allele, respectively. The bar graphs to the right of the graphical genotypes are the phenotypic difference comparison between the HR and HNR pair within each recombinant group (*n *>* *50 in HR and HNR). The allele effects are calculated as the LA (the number of leaves above the primary ear) difference between HR and HNR. Red bars with ** indicate a significant difference at *P *<* *0.01 after Bonferroni correction. Black bars indicate no significant difference between HR and HNR. (b) Severe recombination suppression in the region of *qLA1‐1*. The *x*‐axis and *y*‐axis show the physical and genetic positions on chromosome 1, respectively. The grey and red lines are the genetic maps of maize‐teosinte BC_2_S_3_ population and maize nested association mapping (NAM) population across the region. The dotted lines indicate the *c*. 20‐Mb region with severe recombination suppression. The open triangle indicates the position of the centromere.

In order to further delimit the position of *qLA1‐1*, an F_2_ population containing 1200 plants was created by self‐fertilizing the HIF that only segregated for *qLA1‐1* (Fig. S1). The two markers that flank *qLA1‐1*, M1 and M7, were first used to identify recombinants from the F_2_ population. A total of 37 recombinants were identified. Five additional markers were then developed to further resolve the breakpoints of the identified recombinants (Table S3). Figure 7(a) shows the high‐resolution map of the F_2_ population. Remarkably, the recombination rate varied substantially across the region. A total of 29 recombinants was identified between markers M1 and M3, which are only *c*. 4 Mb apart, and eight recombinants were identified in the *c*. 3.5‐Mb region between M6 and M7. By contrast, no recombinants were observed between M3 and M6, which are *c*. 20 Mb apart, suggesting the presence of severe recombination suppression over this region.

Based on the marker genotypes across the region, the 37 recombinants were divided into six genotype groups. Owing to the limited field space of the Beijing trial in 2014, for each genotype group, only one recombinant family containing > 300 F_3_ seeds was selected and planted in Beijing for phenotypic testing. Within each F_3_ family, HR and HNR plants were identified using appropriate markers and the four leaf number and flowering time traits were evaluated. Following the fine‐mapping procedure of Huang *et al*. (2015), *qLA1‐1* was finally delimited to the region between marker M2 and M7 that includes the 20‐Mb recombination suppression region between M3 and M6 described earlier. This severe recombination suppression prevented us from further refining the causal region of *qLA1‐1*. We checked the recombination rate of the corresponding region in the genetic map of the maize nested association mapping (NAM) population. Interestingly, the recombination rate over the region appears to be normal in the maize NAM map (Fig. 7b).

## Discussion

**Figure 8:**
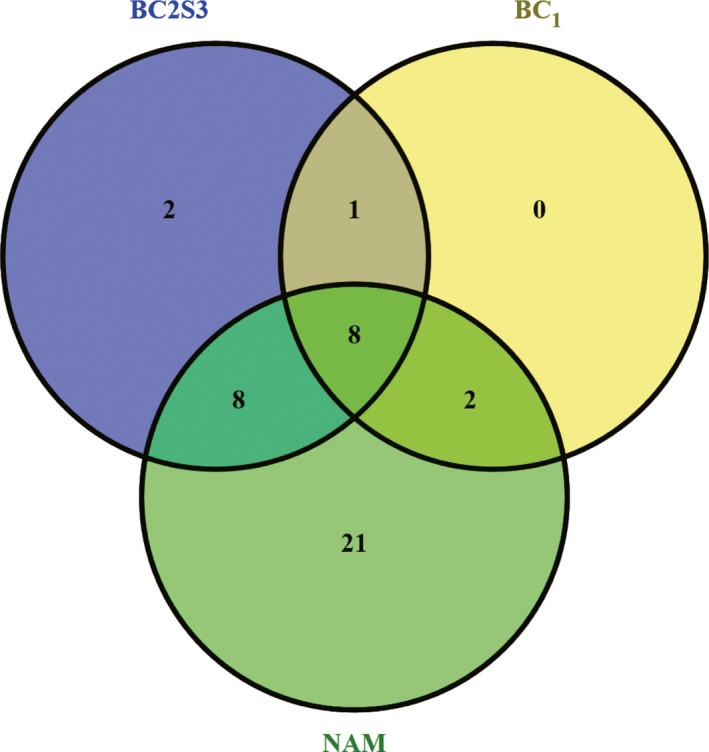
The congruence of quantitative trait loci (QTLs) identified across studies. BC_2_S_3_ represents the maize‐teosinte recombinant inbred line population used in this study. Nested association mapping (NAM) and BC_1_ represent the previous two studies by Buckler *et al*. (2009) and Briggs *et al*. (2007), respectively.

In this study, using a large maize‐teosinte BC_2_S_3_ recombinant inbred line (RIL) population, we conducted comprehensive quantitative trait locus (QTL) mapping for four leaf number and flowering time traits and investigated their genetic relationships. To evaluate the congruence of the identified QTLs, we compared the QTLs detected in this BC_2_S_3_ population to those reported in previous studies. Few studies have specifically dissected the genetic basis of leaf number and its components leaves above (LA) and below (LB) the primary ear; we thus limited our comparisons to two previous large flowering time mapping studies. One is the largest flowering time studies that employed the maize nested association mapping (NAM) population (Buckler *et al*., 2009). The other is the study of Briggs *et al*. (2007) in which QTL mapping for flowering time was conducted in a large maize‐teosinte BC_1_ population. Of 19 QTLs for days to anthesis (DTA) identified in this BC_2_S_3_ population, 16 (84%) were also detected in the maize NAM population (Fig. 8). Briggs *et al*. (2007) identified 11 QTLs for DTA in a maize‐teosinte BC_1_ population, of which nine (81.8%) were detected in our study (Fig. 8). These results suggest that these flowering time QTLs are stably expressed in intraspecies and interspecies populations. The high QTL correspondence indicates that this maize‐teosinte BC_2_S_3_ population is a representative and powerful tool to assess the genetic architectures of complex traits, as indicated in previous studies that used this population (Hung *et al*., 2012; Lin *et al*., 2012; Wills *et al*., 2013; Lang *et al*., 2014; Huang *et al*., 2015).

In maize, the total number of leaves can be specifically partitioned into two components according to the position of the primary ear, LA and LB. LA and LB are two important selection parameters in plant architecture breeding because an optimal proportion of LA and LB is critical for improving plant architecture to maximize the photosynthetic potential of plant populations. Despite this importance, few studies have specifically dissected the genetic basis of LA and LB. The phenotypic analysis demonstrated that LA and LB exhibit very low phenotypic correlation, consistent with observations in other maize populations (X. F. Wang & F. Tian, unpublished). The QTL mapping results revealed contrasting genetic architectures for LA and LB. LA was controlled by a large‐effect QTL plus many small‐effect QTLs, whereas LB was controlled by numerous small‐effect QTLs. Furthermore, only three QTLs were simultaneously detected for LA and LB, suggesting that LA and LB tend to be under relatively independent genetic control. The analysis of the positive or negative effect of QTLs for LA and LB demonstrated that the maize alleles at LA QTLs tend to increase the number of leaves above the primary ear, whereas the maize alleles at LB QTLs tend to decrease the number of leaves below the primary ear, indicating that LA and LB might be under differential directional selection during maize domestication and improvement. This locus‐level directional selection might reflect the footprint of past breeding selection for an optimal proportion of LA and LB. The source of photosynthate for grain filling is largely from leaves at and above the ear‐node; therefore, plants with more leaves above the primary ear potentially have higher photosynthetic potential owing to the enhanced source strength, which allows greater accumulation and transport of dry matter into the sink during grain filling (Shaver, 1983; Na & Haiqiu, 2006). Appropriately reducing the number of leaves below the primary ear can lower the ear height, thus enhancing the lodging resistance of the population. Importantly, the genetic independence between LA and LB provides the potential for independent genetic manipulation of LA and LB for ideal plant architecture breeding.

The genetic analyses of TLN, LA and LB provide an important example for demonstrating the genetic relationship between complex traits and their components. Of 34 nonoverlapping leaf number QTLs, the majority exhibited effects on TLN and one of its components, nine QTLs were specific to either LA or LB, and three QTLs had effects on both LA and LB. No QTLs that affected all three leaf number traits were detected. This mapping difference is mostly due to the opposite QTL effect direction of LA and LB and illustrates the necessity of partitioning a complex trait to its corresponding components of biological or agronomic importance; otherwise, the specific component effects might be missed, particularly when the components are under different evolutionary or selective forces, as in LA and LB.

The genetic dissection of leaf number and flowering time traits indicated that *c*. 40% of total QTLs showed pleiotropic effects on leaf number and flowering time. Of these shared loci, 73.3% of QTLs involved effects from LB, whereas only 26.7% involved effects from LA. These results suggest that leaf number and flowering time are commonly regulated at a moderate level overall and that the genetic overlap mostly results from genetic sharing between LB and DTA. The flowering time of a maize plant is determined largely by the timing of floral transition and subsequent developmental processes that affect the timing of pollen shed and silk exertion, whereas the number of leaves that a maize plant produces is determined by the rate and pattern of leaf initiation during the vegetative stage, and the duration of vegetative development which is controlled mainly by the timing of floral transition. Mutants with delayed floral transition usually produce more leaves as a result of extended vegetative leaf initiation, as observed for several previously cloned flowering time genes in maize (Colasanti *et al*., 2006; Muszynski *et al*., 2006). Genes that affect the rate and pattern of leaf initiation can change the leaf number and their arrangements on plants by altering the spatial (phyllotaxy) and temporal (plastochron) patterns of leaf initiation (Veit *et al*., 1998; Jackson & Hake, 1999; Giulini *et al*., 2004; Chuck *et al*., 2010, 2014). For example, *aberrant phyllotaxy1* (*abph1*) specifically affects phyllotaxy and its loss‐of‐function mutation initiates leaves in a decussate pattern, therefore doubling the total number of leaves per plant (Jackson & Hake, 1999; Giulini *et al*., 2004). *SBP‐box* genes *ub2*,* ub3* and *tsh4* function redundantly to repress leaf initiation. Their double or triple loss‐of‐function mutants resulted in a shortened plastochron and consequently an increased number of leaves. Despite the increase in leaf number, the double or triple mutants flowered at the same time as the wild‐type plants. Given these developmental and regulatory differences, it is reasonable that leaf number and flowering time are commonly regulated at a moderate level. It is largely unclear how LA and LB are independently regulated in development and why LB rather than LA tends to be commonly regulated with DTA. Leaf number is an important intrinsic signal of floral induction (Colasanti & Muszynski, 2009), but how the signal is elaborately coordinated with floral transition, particularly the conversion of the upper axillary meristems into ear primordia, is unclear. Maize plants have developed sophisticated genetic mechanisms that specify the compartments of the phytomer during each developmental stage. Future genetic and developmental studies are required to clarify the underlying mechanisms.

Maize was domesticated in southern Mexico from its wild ancestor, teosinte (*Zea mays* ssp. *parviglumis*), a species that requires short days to flower (Matsuoka *et al*., 2002). This ancestral photoperiod sensitivity was gradually lost during the adaptation of maize to higher latitudes of temperate environments. The analysis of the allele effect direction of flowering time QTLs demonstrated that the maize alleles at all DTA QTLs are associated with early flowering (Fig. S3b), suggesting that DTA may be under strong directional selection during maize adaptation to temperate environments. We further demonstrated that the previously cloned genes *ZmCCT*,* ZCN8* and *dlf1*, are all located at the peaks of DTA and leaf number QTLs. Although *ZmCCT* was cloned as a flowering time QTL and shown to be involved in the post‐domestication adaptation of maize (Hung *et al*., 2012; Yang *et al*., 2013), *ZCN8* and *dlf1* were initially determined to function during floral transition based on expression or mutant analysis. Our study demonstrates that the natural variation at *ZCN8* and *dlf1* might play important roles in shaping the flowering adaptation of maize. Furthermore, we found that their effects on leaf number were only limited to LB, with no significant effects for LA detected at these three genes. Further population genetic analyses of *ZCN8*,* dlf1* and *ZmCCT* may provide important insights into how these genes interact to shape maize local adaptation.

In addition to the common QTLs for leaf number and flowering time, QTLs that specifically affect leaf number or DTA were identified. Among them, *qLA1‐1* on chromosome 1 is a major‐effect QTL that specifically affects LA, and was further validated via NIL analysis. Through fine mapping, we delimited *qLA1‐1* to a region with severe recombination suppression, which prevented us from further refining the causal region. Interestingly, the recombination rate of corresponding region in the genetic map of the maize NAM population appears to be normal, suggesting that the recombination suppression at *qLA1‐1* might be derived from teosinte. Fang *et al*. (2012) reported a *c*. 50‐Mb inversion on the short arm of chromosome 1 that is *c*. 80 Mb upstream of the *qLA1‐1* region. This *c*. 50‐Mb inverted arrangement was found to dramatically suppress recombination (Fang *et al*., 2012) (Fig. 7b). We hypothesize that the recombination suppression observed in the *qLA1‐1* region might also be associated with a large structural variation from teosinte. Further genetic and cytological experiments are needed to confirm this hypothesis. It also remains unclear at this time how *qLA1‐1* specifically affects LA without influencing LB and flowering time. The final cloning of *qLA1‐1* will provide important insights into the underlying regulatory mechanisms. Owing to the effect specificity of *qLA1‐1* on LA, *qLA1‐1* holds promise for use as a tool to improve the photosynthetic potential of canopy architecture without changing LB and flowering time.

## Author contributions

F.T. and D.L. designed the research. D.L. and X.W. performed the data analysis. D.L., X.W., X.Z., Q.C., G.X., D.X., C.W., Y.L., L.W., C.H., J.T. and Y.W. conducted fieldwork. F.T. and D.L. wrote the manuscript.

## Supporting information

Please note: Wiley Blackwell are not responsible for the content or functionality of any supporting information supplied by the authors. Any queries (other than missing material) should be directed to the *New Phytologist* Central Office.

**Fig. S1 **Graphical genotypes of a heterogeneous inbred family (HIF) in maize‐teosinte BC_2_S_3_ population used for fine mapping *qLA1‐1*.
**Fig. S2 **Candidate gene for *qLB2‐3* on chromosome 2.
**Fig. S3 **The additive effect of each quantitative trait locus (QTL) for total leaf number (TLN) and days to anthesis (DTA).
**Table S1 **Quantitative trait loci (QTLs) for leaf number and flowering time identified in a maize‐teosinte BC_2_S_3_ recombinant inbred line (RIL) population
**Table S2** Quantitative trait locus (QTL) correspondence likelihood expected by chance
**Table S3** The primer sequences of markers used for near‐isogenic line (NIL) analysis and *qLA1‐1* fine mapping
