# Supplementary material for: The genetic architecture of leaf number and its genetic relationship to flowering time in maize
Source: New Phytol. 2015 Nov 23;210(1):256–68. doi: 10.1111/nph.13765 (PMC5063108; doi:10.1111/nph.13765)
Supplement: Supplementary file 1 — Fig. S1 Graphical genotypes of a heterogeneous inbred family (HIF) in maize‐teosinte BC2S3 population used for fine mapping qLA1‐1. Fig. S2 Candidate gene for qLB2‐3 on chromosome 2. Fig. S3 The additive effect of each quantitative trait locus (QTL) for total leaf number (TLN) and days to anthesis (DTA). Table S1 Quantitative trait loci (QTLs) for leaf number and flowering time identified in a maize‐teosinte BC2S3 recombinant inbred line (RIL) population Table S2 Quantitative trait locus (QTL) correspondence likelihood expected by chance Table S3 The primer sequences of markers used for near‐isogenic line (NIL) analysis and qLA1‐1 fine mapping [file NPH-210-256-s001.pdf]

New Phytologist

New Phytologist Supporting Information Figs S1–S3 and Tables S1–S3

Article title: The genetic architecture of leaf number and its genetic relationship to flowering time in maize

Authors: Dan Li, Xufeng Wang, Xiangbo Zhang, Qiuyue Chen, Guanghui Xu, Dingyi Xu, Chenglong Wang, Yameng Liang, Lishuan Wu, Cheng Huang, Jinge Tian, Yaoyao Wu and Feng Tian

Article acceptance date: 15 October 2015

The following Supporting Information is available for this article:

Fig. S1 Graphical genotypes of a heterogeneous inbred family (HIF) in maize-teosinte BC_2S_3 population used for fine mapping *qLA1-1*.

Fig. S2 Candidate gene for *qLB2-3* on chromosome 2.

Fig. S3 The additive effect of each quantitative trait locus (QTL) for total leaf number (TLN) and days to anthesis (DTA).

Table S1 Quantitative trait loci (QTLs) for leaf number and flowering time identified in a maize-teosinte BC_2S_3 recombinant inbred line (RIL) population

Table S2 Quantitative trait locus (QTL) correspondence likelihood expected by chance

Table S3 The primer sequences of markers used for near isogenic line (NIL) analysis and *qLA1-1* fine mapping

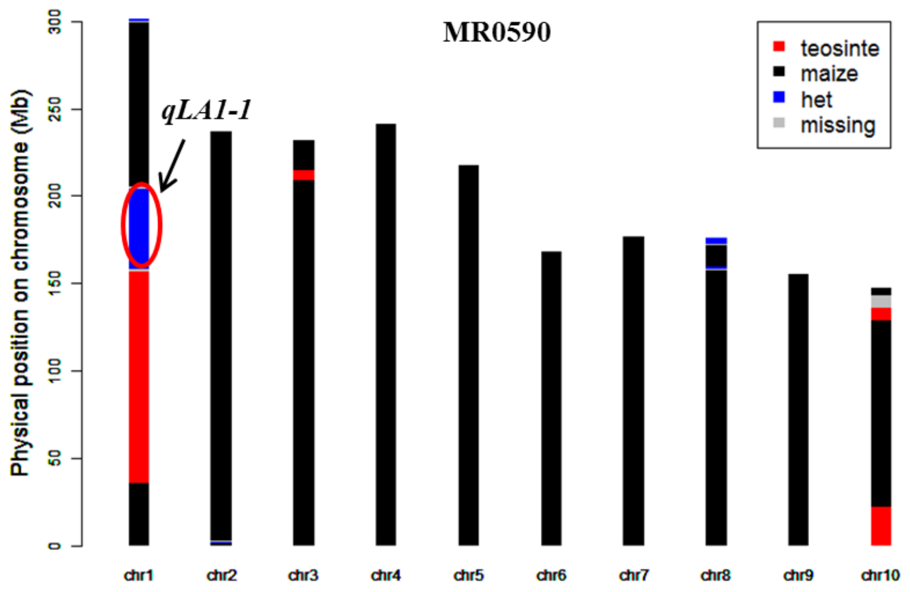

Fig. S1 Graphical genotypes of a heterogeneous inbred family (HIF) used for fine mapping *qLA1-1***.** Black box indicates regions homozygous for the W22; red box indicates regions homozygous for the teosinte; blue box indicates heterozygous regions; grey box indicates unknown regions. The heterozygous region at *qLA1-1* is indicated by red cycle. LA, the number of leaves above the primary ear.

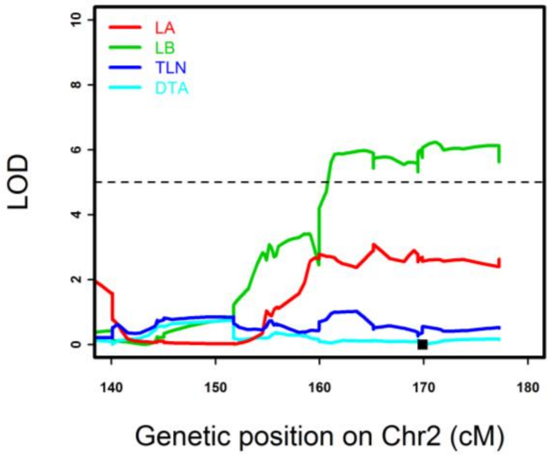

Fig. S2 Candidate gene for *qLB2-3* on chromosome 2. The *x*-axis shows the genetic position along the chromosome 2. The *y*-axis represents the logarithm of odds (LOD) score of each scanning position. Different colored lines indicate different traits. The dotted line represents the threshold of claiming significant quantitative trait locus (LOD = 5). The black square box above the y-axis indicates the position of *ZAP1*. LA, the number of leaves above the primary ear; LB, the number of leaves below the primary ear; TLN, total leaf number; DTA, days to anthesis.

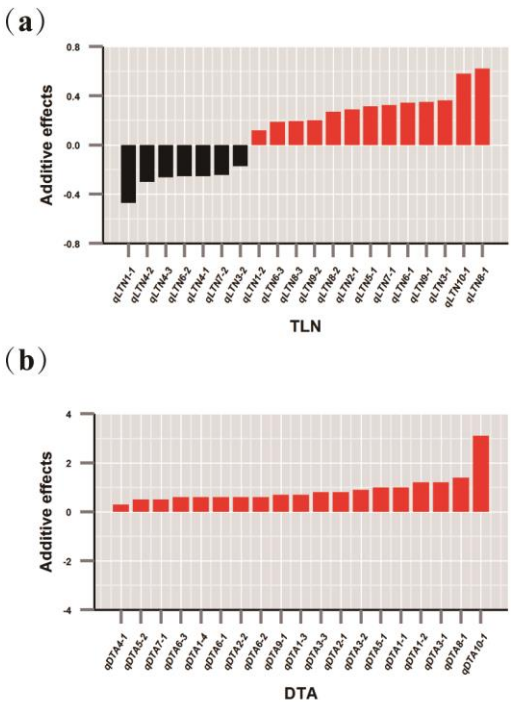

Fig. S3 The additive effect of each quantitative trait locus (QTL) for total leaf number (TLN) and days to anthesis (DTA). The additive effect distribution of QTLs for (a) TLN and (b) DTA. The *x*-axis indicates the name of each QTL and the *y*-axis indicates the additive effect of the teosinte allele relative to the maize allele at each QTL.

Table S1 Quantitative trait loci (QTLs) for leaf number and flowering time identified in maize-teosinte BC_2S_3 recombinant inbred line (RIL) population

Traits	Chr	QTL	LOD	Var (%)	Additive effect	Selection Features	Left position (cM)	Peak position (cM)	Right position (cM)
LA	1	*qLA1-1*	89.8	21.5	-0.42	√	91.2	95.2	95.3
LA	1	*qLA1-2*	12.1	2.3	-0.12	√	43.0	47.1	47.8
LA	1	*qLA1-3*	5.5	1.0	0.09	√	2.2	5.3	9.2
LA	2	*qLA2-1*	7.4	1.4	-0.11	√	24.8	27.2	48.3
LA	3	*qLA3-1*	5.6	1.1	-0.10	√	93.4	97.2	138.1
LA	4	*qLA4-1*	12.5	2.4	-0.15	√	73.6	75.1	77.5
LA	4	*qLA4-2*	21.4	4.2	-0.20	√	89.3	90.8	95.3
LA	5	*qLA5-1*	23.9	4.7	-0.12	√	77.8	82.5	86.3
LA	5	*qLA5-2*	14.1	2.7	-0.16	√	109.0	110.7	111.5
LA	6	*qLA6-1*	24.4	4.8	-0.17		105.5	105.9	106.5
LA	6	*qLA6-2*	18.2	3.6	-0.13	√	11.5	14.1	16.5
LA	8	*qLA8-1*	11.3	2.2	0.10	√	123.8	124.7	127.1
LA	8	*qLA8-2*	5.5	1.0	0.08	√	54.1	62.6	66.2
LA	9	*qLA9-1*	5.6	1.0	-0.07	√	2.2	7.1	21.0
LA	10	*qLA10-1*	5.5	1.0	0.06		56.1	79.9	83.3
LB	1	*qLB1-1*	12.9	2.2	0.21	√	33.5	33.7	34.8
LB	2	*qLB2-1*	12.8	2.1	0.30	√	65.7	70.7	75.9
LB	2	*qLB2-2*	7.8	1.3	0.22		20.2	22.2	25.5
LB	2	*qLB2-3*	6.2	1.0	-0.18	√	160.6	171.2	177.2
LB	3	*qLB3-1*	12.3	2.0	0.21	√	36.6	38.1	39.0
LB	3	*qLB3-2*	11.1	1.8	0.27	√	76.9	77.2	78.3
LB	4	*qLB4-1*	10.6	1.8	-0.16	√	56.4	57.8	66.9
LB	4	*qLB4-2*	6.6	1.1	-0.20	√	124.7	127.5	135.1
LB	5	*qLB5-1*	23.4	4.0	0.27	√	79.5	81.7	82.9
LB	5	*qLB5-2*	16.6	2.8	0.29		17.0	19.5	22.0
LB	6	*qLB6-1*	26.8	4.6	0.38		78.4	78.8	79.6
LB	6	*qLB6-2*	10.4	1.7	0.26	√	97.0	99.1	108.0
LB	7	*qLB7-1*	33.9	6.0	0.33	√	148.0	149	150.1
LB	7	*qLB7-2*	9.1	1.5	-0.19	√	27.0	32.9	37.5
LB	8	*qLB8-1*	18.0	3.0	0.40	√	77.2	77.6	78.3
LB	8	*qLB8-2*	13.5	2.3	0.28	√	88.7	89.6	90.2
LB	8	*qLB8-3*	11.1	1.8	0.23		43.2	44.7	45.7
LB	9	*qLB9-1*	20.3	3.5	0.34	√	66.2	71.4	72.5
LB	10	*qLB10-1*	18.4	3.1	0.43	√	37.8	39.4	40.9
TLN	1	*qTLN1-1*	30.7	5.5	-0.47	√	92.4	95.2	95.3
TLN	1	*qTLN1-2*	5.7	1.0	0.12	√	7.6	16.6	29
TLN	2	*qTLN2-1*	10.9	1.9	0.29	√	66.4	70.7	75.9

TLN	3	*qTLN3-1*	19.3	3.4	0.36	√	46.0	47.9	49.6
TLN	3	*qTLN3-2*	5.6	0.9	-0.17	√	79.2	133.9	136.6
TLN	4	*qTLN4-1*	8.5	1.4	-0.25	√	77.7	89.3	89.8
TLN	4	*qTLN4-2*	15.6	2.7	-0.30	√	56.4	57.3	59.0
TLN	4	*qTLN4-3*	9.5	1.6	-0.26	√	124.6	127	135.0
TLN	5	*qTLN5-1*	15.3	2.6	0.31		16.5	18.7	20.5
TLN	6	*qTLN6-1*	21.6	3.8	0.34	√	78.5	79.2	81.3
TLN	6	*qTLN6-2*	12.3	2.1	-0.25	√	15.4	16.5	19.0
TLN	6	*qTLN6-3*	5.4	0.9	0.19	√	38.0	47	57.8
TLN	7	*qTLN7-1*	24.8	4.4	0.33	√	146.5	148	148.2
TLN	7	*qTLN7-2*	10.8	1.8	-0.24	√	17.1	19.5	27.5
TLN	8	*qTLN8-1*	51.3	9.8	0.62		77.6	77.6	78.2
TLN	8	*qTLN8-2*	12.5	2.1	0.27		44.0	45.3	45.8
TLN	8	*qTLN8-3*	12.2	2.1	0.20	√	112.7	119.7	124.2
TLN	9	*qTLN9-1*	17.6	3.1	0.35		65.1	65.7	67.9
TLN	9	*qTLN9-2*	8.4	1.4	0.20	√	100.5	106.9	119.0
TLN	10	*qTLN10-1*	26.4	4.7	0.58	√	38.5	39.4	40.9
DTA	1	*qDTA1-1*	15.2	2.9	1.0	√	84.2	87.3	87.5
DTA	1	*qDTA1-2*	14.7	2.8	1.2	√	139.3	140.5	141.7
DTA	1	*qDTA1-3*	8.8	1.6	0.7	√	7.4	8.6	11.5
DTA	1	*qDTA1-4*	8.3	1.5	0.6	√	30.5	34.2	34.8
DTA	2	*qDTA2-1*	12.7	2.4	0.8	√	19.5	23.9	36.5
DTA	2	*qDTA2-2*	6.7	1.2	0.6	√	65.5	66.5	68.5
DTA	3	*qDTA3-1*	22.8	4.4	1.2		107.6	108.5	109.0
DTA	3	*qDTA3-2*	11.7	2.2	0.9	√	75.0	75.5	76.9
DTA	3	*qDTA3-3*	7.9	1.5	0.8	√	47.2	53.0	54.8
DTA	4	*qDTA4-1*	7.5	1.4	0.3	√	149.1	152.4	155.5
DTA	5	*qDTA5-1*	18.5	3.5	1.0	√	15.0	18.7	20.5
DTA	5	*qDTA5-2*	6.3	1.2	0.5	√	58.1	59.4	67.0
DTA	6	*qDTA6-1*	9.4	1.7	0.6	√	106.0	108.9	110.4
DTA	6	*qDTA6-2*	7.7	1.4	0.6	√	78.6	80.4	82.0
DTA	6	*qDTA6-3*	6.1	1.1	0.6	√	44.4	56.6	60.5
DTA	7	*qDTA7-1*	8.1	1.5	0.5	√	146.0	148.0	150.0
DTA	8	*qDTA8-1*	33.9	6.7	1.4	√	77.2	77.6	77.6
DTA	9	*qDTA9-1*	9.8	1.8	0.7	√	64.4	66.6	80.4
DTA	10	*qDTA10-1*	74.5	16.6	3.1	√	39.6	40.6	40.8

The left and right positions are the 2-LOD support intervals of each QTL. Positive and negative additive effects indicate that the teosinte allele increase and decrease the values of the phenotypes, respectively. √, QTL region overlaps with previously identified selection features by Hufford *et al*. (2012). LA, the number of leaves above the primary ear; LB, the number of leaves below the primary ear; TLN, total leaf number; DTA, days to anthesis.

New Phytologist

Hufford MB, Xu X, Van Heerwaarden J, Pyhäjärvi T, Chia J-M, Cartwright RA, Elshire RJ, Glaubitz JC, Guill KE, Kaeppler SM. 2012. Comparative population genomics of maize domestication and improvement. *Nature Genetics* **44:** 808–811.

Table S2 Quantitative trait locus correspondence likelihood expected by chance

	**LA**	**LB**	**TLN**
**LB**	0.1344	-	-
**TLN**	0.0017	4.83E-08	-
**DTA**	0.0414	2.28E-08	4.83E-08

LA, the number of leaves above the primary ear; LB, the number of leaves below the primary ear; TLN, total leaf number; DTA, days to anthesis.

Table S3 The primer sequences of markers used in near isogenic line analysis and *qLA1-1* fine mapping

Marker	Type	Chr.	Forward primer	Reverse primer
M1	InDel	1	CTTCGAGCACTTCCTTCACC	ATCATGGGTTTCCAGAGGC
M2	InDel	1	TCTGACTGAATGCCAACC	TAGCCTTTCCCTCACCAG
M3	InDel	1	AGATGGGAGCCTTGGAAGAT	CCTAGCAACTCTGTATTGTATTGTATT
M4	InDel	1	TCCCGTTCCTCACCCGATT	CACCGATAAACAACACACCG
M5	InDel	1	ATACCGCCGCACAGTAC	CTACAACAACGGGCAGTG
M6	InDel	1	ATACTGCTTGTTGGGCTTGG	TCTACTACTGCTACGTCGGC
M7	InDel	1	CACCACAACCACCTTTCC	TCAGTTCCCTTCCACGAC
dlf1	InDel	1	TGTCCCCTCCACGTCTATAA	CCCAAATATCTTTAGTATCGGTTC
ZCN8	InDel	8	TTCCCACCACCTTCTAGCTG	CATGAGGCCAGTAGACACCA
ZMCCT	InDel	10	CACCCGTAGCCAATAACTTG	CCCAAATATCTTTAGTATCGGTTC

LA, the number of leaves above the primary ear.
